# Gifts to Christ

**Published:** 1890-01-18

**Authors:** 


### Words of Consolation.

### GIFTS TO CHRIST.

*(For Reading to the Sick.)*

Many hundred years before the birth of Christ, the Jewish prophets foretold that in Him should *all* the families of the earth be blessed; and on the sinless babe's presentation to the Father in the Temple when He was eight days old, the aged Simeon, taking Him into his arms, blessed Him as a light which had come to lighten the Gentiles and to be the glory of His people Israel. To mark the former promise more emphatically, it happened about this time that certain wise men, or kings, as they are sometimes called, appeared in Jerusalem enquiring where the King of the Jews should be born. They had been led by a star from far distant lands, following it across trackless deserts, to worship at the throne of the God King, whom they hoped to find at the end of their journey.

Whether they had suddenly lost sight of their heavenly guide, or their faith had failed them in the noise and confusion of a large city, we know not, but they began to ask their way just as they had arrived at the end of their quest. These enquiries led to other results than they anticipated. Herod, by the help of the chief priests and scribes, was able to tell them that the Christ should be born in Bethlehem of Judah. Comforted by the information they had received, the wise men went on with renewed vigour until the star stood over the place where the young child lay.

Do we follow the example of these wise and good men? Do we think over the blessings which God showers around our daily life? Do we study Him in nature, and long to know more of One so great and wonderful? These wise men most probably considered or studied the heavens, the works of God's fingers, the moon and the stars which He had created. They "looked through nature up to nature's God," and exclaimed with the Psalmist, "O Lord, our Governor, how excellent is Thy name in all the world, Thou that hast set Thy glory above the heavens." We will follow their example and seek for the Saviour with all our hearts. We will leave our homes, if necessary, and our dearest ties to worship Him with our bodies. We will bring whatsoever gifts we may have to do Him service. We may have little or no gold to offer, but we can give truth and purity to His honour, which are priceless treasures, adding the incense of praise to His glory, and the myrrh of self-denial in our narrow daily path.

But where shall we find the Child Christ, say you? On every bed of sickness, in every lowly home, in every abode of ignorance and vice. In all these places we may seek and find Him.

